# Case Report: Hybrid treatment of a giant V1 segment vertebral artery aneurysm with bypass and subclavian artery stent

**DOI:** 10.3389/fsurg.2026.1867301

**Published:** 2026-08-12

**Authors:** Chao Yan, Jinren Zhou, Yingming Xu, Yifeng Pan, Zhiwei Gao

**Affiliations:** 1 Department of Vascular Surgery, The Second Affiliated Hospital of Zhejiang University School of Medicine, Hangzhou, China; 2 Vascular Interventional Surgery, XingYi People's Hospital, XingYi, GuiZhou, China

**Keywords:** hybrid treatment, jugular vein graft, patency, subclavian artery stent, vertebral artery aneurysm

## Abstract

Although rare, an exploration of treatment strategies for V1 segment vertebral artery aneurysms is warranted. Herein, we present the case of a 32-year-old woman diagnosed with a left supraclavicular region vertebral artery aneurysm treated at our centre. Computed tomography conducted post-admission revealed a thrombosed giant aneurysm measuring 69 × 37 mm. A hybrid repair was performed, combining a carotid artery-jugular vein graft-vertebral artery bypass with subclavian artery stent implantation. No neurological symptoms were observed. Stent and bypass patency were maintained at the 11-month follow-up. Overall, this case suggests that hybrid repair offers a less invasive and more effective alternative treatment for vertebral artery aneurysms.

## 1 Introduction

Extracranial vertebral artery aneurysms (VAAs) are rare, with vertebral artery lesions occurring in approximately 1% of cases (1). VAAs may develop due to trauma, tumour invasion, or infection. Common clinical symptoms and complications include local compression, dizziness, headache, and a risk of rupture. Vertebral aneurysms in the V1 region are generally less common. Treatment options for VAAs include surgical and endovascular approaches. Open repair combined with endovascular treatment has traditionally been applied for refractory cases involving surgically complex regions.

## 2 Case presentation

A 32-year-old woman with no history of surgery or trauma was admitted to our centre with a mass in the left supraclavicular region. She reported experiencing occasional dizziness in enclosed spaces and an intermittent sense of constriction, but denied any other symptoms of posterior circulatory ischaemia. The patient had no history of hypertension or diabetes, or any family history of aneurysms.

**Figure 1:**
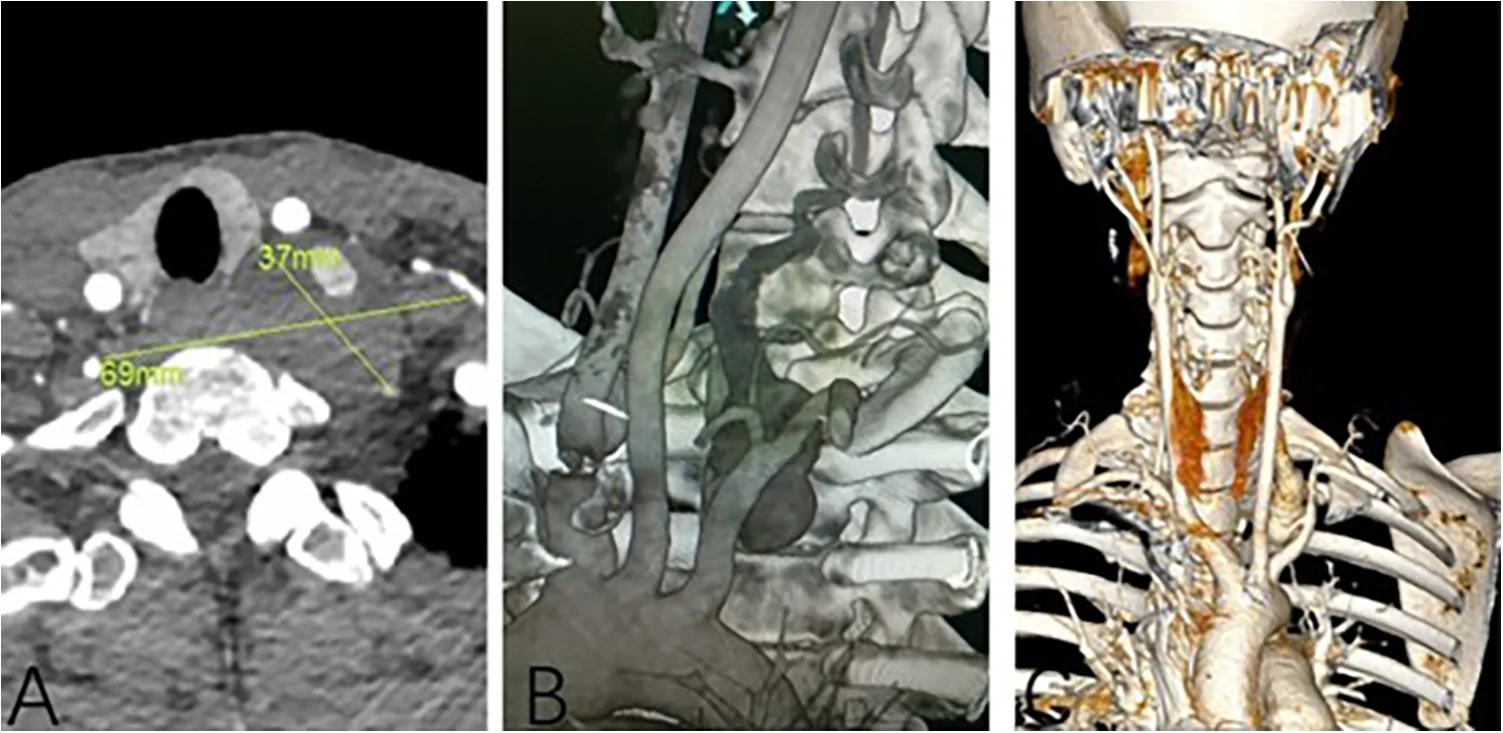
Contrast-enhanced CT **(A)** and 3D-CTA **(B,C)** images showing a thrombosed aneurysm in the V1 segment of left vertebral artery. The cross section shows a 69 × 37 mm thrombosed aneurysm in the left lower neck. CT, computed tomography; 3D-CTA, three-dimensional computed tomography angiography.

Physical examination revealed a pulsatile mass in the supraclavicular region, without any redness or swelling. The rheumatological workup was unremarkable, and neurological tests revealed no abnormalities. However, computed tomography (CT) revealed a giant thrombosed aneurysm in the left supraclavicular region (Figure 1), measuring 69 × 37 mm on CT.

### 2.1 Surgical process

**Figure 2:**
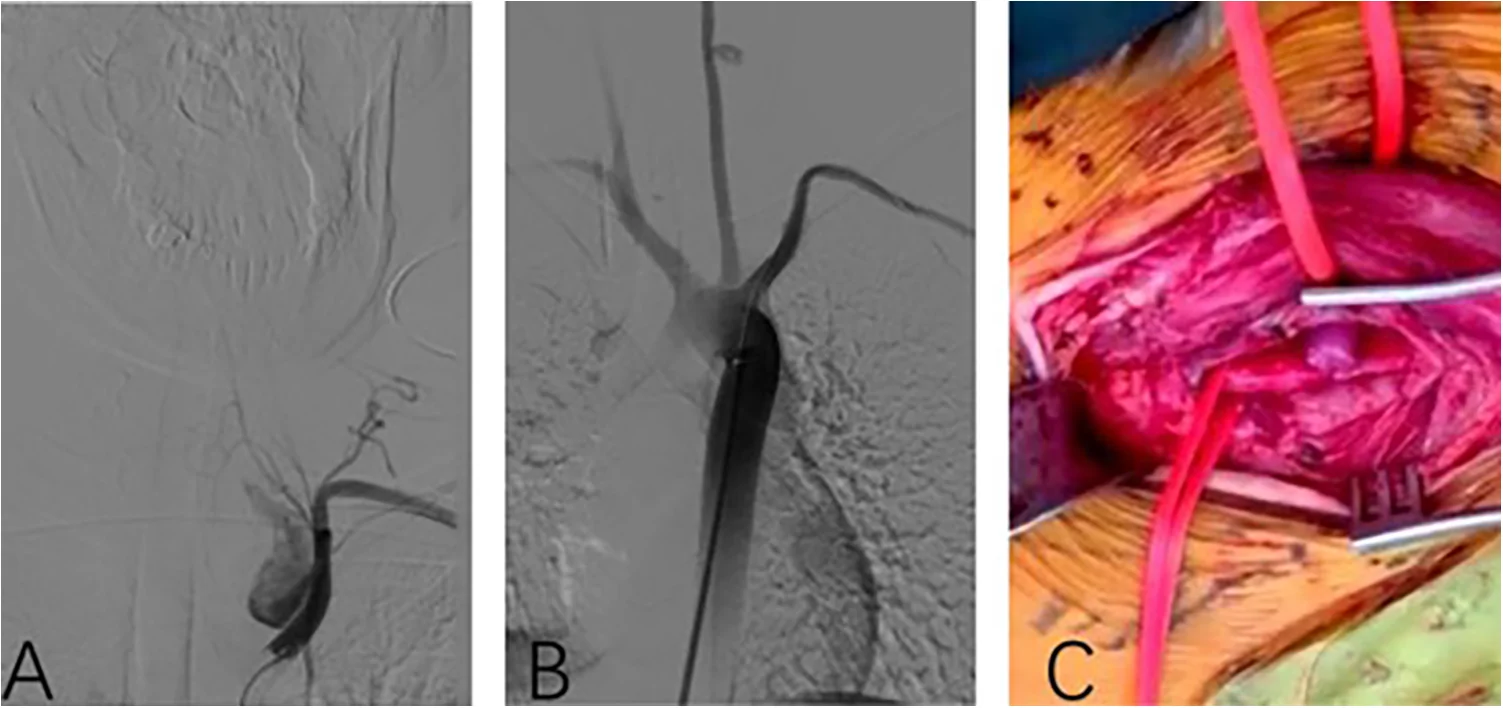
**(A)** Left subclavian angiogram showing a giant aneurysm in the V1 segment and the normal structure of the vertebral artery at the proximal side of the V1 segment. The aneurysm was partially filled with contrast agent. **(B)** Left subclavian angiogram following subclavian stent implantation demonstrating complete obliteration of the aneurysm. **(C)** Distal vertebral-to-internal-carotid bypass with a reversed external jugular vein graft.

Surgery was conducted under general anaesthesia, as follows: An incision measuring approximately 10 cm was made in the left supraclavicular region. The posterior margin of the sternocleidomastoid muscle was gradually separated to reach the deep layer. The giant VAA was located infraclavicularly, with the distal outflow tract extending towards the cervical vertebra, making exposure difficult. Resection of the C6 and C7 vertebrae was conducted to facilitate the partial exposure of the V2 segment of the vertebral artery. The external jugular vein was separated and prepared as an autologous bypass graft. An end-to-side anastomosis was performed between the vertebral and common carotid arteries using the external jugular vein. Suture ligation was conducted on the proximal outflow tract of the vertebral aneurysm. The femoral artery was punctured, and a 5F sheath was subsequently inserted. The subclavian artery was catheterised, and an 8 × 50 mm stent (GORE VIABAHN, USA) was subsequently deployed to cover the origin of the vertebral artery. The stent was well-shaped and demonstrated a good flow rate. Aortic arch angiography was finally performed to assess the patency of the stent and carotid-to-vertebral bypass (Figure 2).

**Figure 3:**
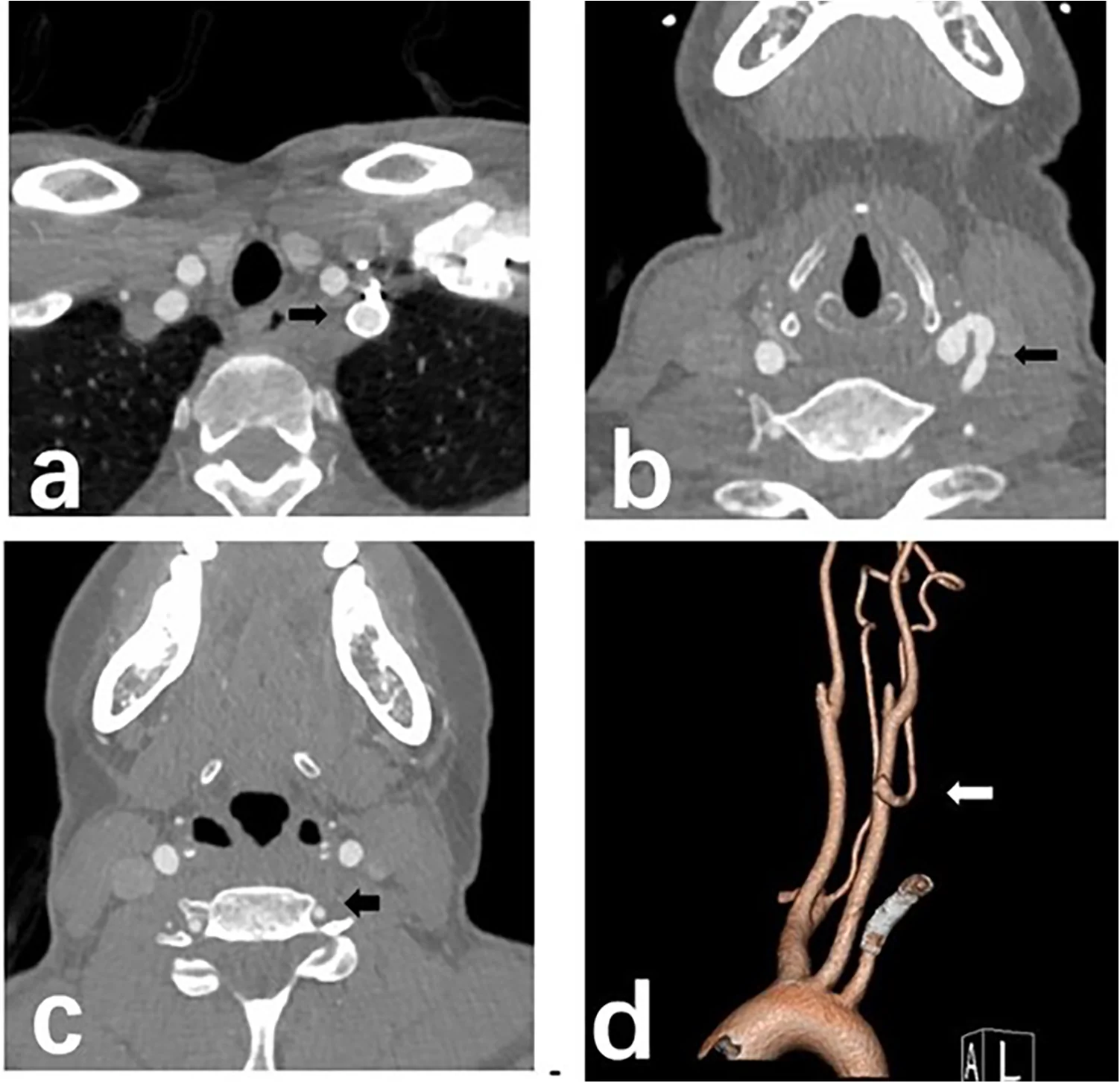
Contrast-enhanced CT and 3D-CTA images obtained 11 months after treatment. **(a)** Left subclavian angiogram showing subclavian artery stent patency. **(b)** Distal-vertebral-to-internal-carotid bypass in the cross section. **(c)** The anterior edge of the foramen was partly resected. **(d)** 3D-CTA showing the subclavian artery stent and vertebral-to-internal-carotid bypass.

The patient was ultimately discharged on aspirin (100 mg) on postoperative day 6. Outpatient follow-ups were conducted at 1, 3, and 11 months postoperatively, with no complications observed. Although the patient complained of pain and weakness in the left upper limb at the 1-month follow-up, these symptoms had improved by the second postoperative month. CT results further confirmed a reduction in the size of the thrombosed aneurysm, and the stent and carotid-to-vertebral bypass patency were found to be preserved at the 11-month follow-up (Figure 3).

## 3 Discussion

The optimal treatment strategy for VAA currently remains controversial. VAA rupture can be fatal due to airway occlusion and bleeding, leading many physicians to favour active intervention over conservative management. Garg et al. (2) previously recommended treatment for patients with pseudoaneurysms or neurological symptoms, regardless of size. Initially, open surgery was chosen as the primary treatment; however, this procedure is invasive and involves a high risk of postoperative complications. Conversely, endovascular treatments offer several advantages, such as reduced trauma, less immediate risk (3), and faster recovery, leading to their gradual replacement of traditional open surgery.

Occlusion of the affected artery and revascularisation of the vertebral artery are commonly used to treat aneurysms. Ligation is a primitive method for blocking vertebral artery flow, while embolisation is the most commonly used form of endovascular treatment. Adverse events may occur following occlusion, with the incidence of neurological complications after ligation or embolisation reported to reac as high as 8% (4). Despite this risk, ligation remains a viable option for emergency operations. The endovascular procedure typically involves both distal and proximal occlusion. However, pure proximal occlusion is commonly performed to avoid any distal embolic complications (5, 6). Additional embolisation options, such as microvascular plug insertion, are also available (7).

Sacrificing the vertebral artery poses a significant risk of posterior circulation ischaemia, making stent grafts or revascularisation the preferred treatment. Revascularisation methods include distal vertebral-to-internal carotid transposition, carotid-to-vertebral bypass, and autologous-external carotid-to-vertebral bypass (8). Stent implantation further provides an alternative treatment which can preserve the patency of the treated artery (9, 10). Patency has been demonstrated in several cases of stent implantation during follow-up.

Aneurysms located at the vertebral artery origin often require invasive direct surgery, potentially involving thoracotomy. Advancements in endovascular therapy seem to have several significant advantages over direct surgery, although this technique still has some limitations. Aortic arch anatomy and the degree of vertebral artery distortion determine the success of stent implantation. Furthermore, covered stents carry a risk of occlusion (11).

**Figure 4:**
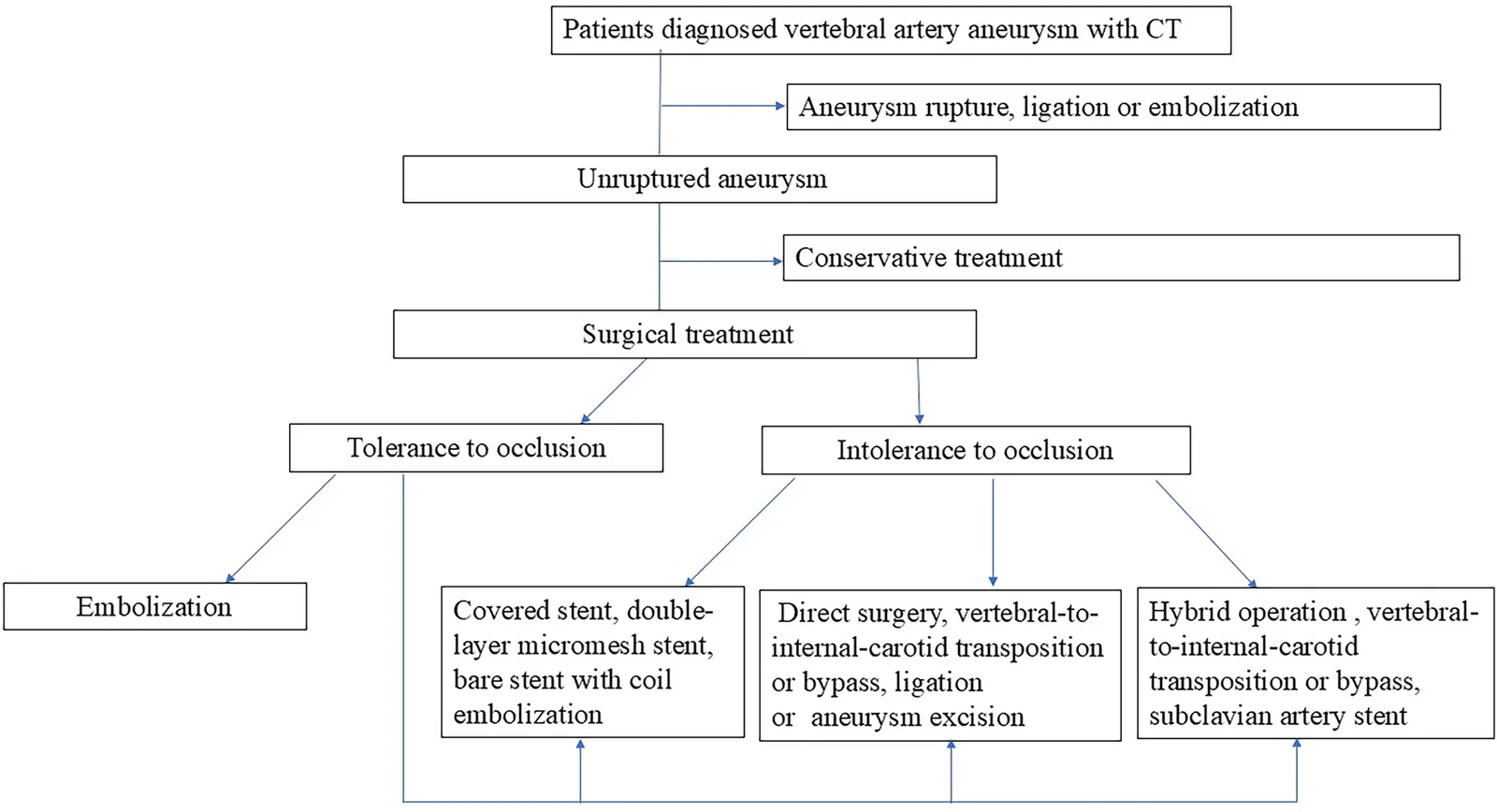
Therapeutic algorithm for V1 segment VAA.

In the present case, the patient was reluctant to undergo thoracotomy due to the invasive nature of this procedure. Given the size and location of the giant VAA, we evaluated the risk of nerve damage. Additionally, a suitable covered stent was not available. Hybrid repair, a less invasive alternative commonly used for subclavian artery aneurysms, was also considered (12, 13). Ultimately, the patient was satisfied with the results of hybrid surgery. This case highlights the importance of individualised management for VAA, suggesting that hybrid surgery is worth exploring as a viable treatment strategy (Figure 4).

In the present case, the VAA was so large that it invaded the cervical vertebrae, leaving insufficient space to perform carotid-to-vertebral artery bypass. Consequently, we sought the assistance of orthopaedic surgeons to resect a portion of the intervertebral foramen. Although the saphenous vein is commonly used in carotid–vertebral artery bypass (14), we opted to use the external jugular vein as the bypass graft owing to its accessibility.

To the best of our knowledge, this is the first case report in the literature in which a VAA was treated with a carotid artery-external jugular vein-vertebral artery bypass in the V2 segment combined with subclavian artery stent implantation. The surgery was completed without complications, highlighting this approach as a viable and promising alternative for managing VAAs.

## 4 Conclusions

Herein, we present the case of a thrombosed giant aneurysm treated with a hybrid procedure. The treatment involved an external jugular vein graft bypass from the carotid artery to the V2 segment of the vertebral artery along with subclavian artery stent implantation. This case demonstrates a less invasive and effective approach for managing VAAs.

## Data Availability

The original contributions presented in the study are included in the article/Supplementary Material, further inquiries can be directed to the corresponding author.
